# Evidence of Subclinical mtDNA Alterations in HIV-Infected Pregnant Women Receiving Combination Antiretroviral Therapy Compared to HIV-Negative Pregnant Women

**DOI:** 10.1371/journal.pone.0135041

**Published:** 2015-08-06

**Authors:** Deborah M. Money, Emily C. Wagner, Evelyn J. Maan, Tessa Chaworth-Musters, Izabelle Gadawski, Julie E. van Schalkwyk, John C. Forbes, David R. Burdge, Arianne Y. K. Albert, Zoe Lohn, Hélène C. F. Côté, The Oak Tree Clinic Research Group

**Affiliations:** 1 Department of Obstetrics and Gynaecology, Faculty of Medicine, University of British Columbia, Vancouver, BC, Canada; 2 Women’s Health Research Institute, BC Women’s Hospital and Health Centre, Vancouver, BC, Canada; 3 Oak Tree Clinic, BC Women’s Hospital and Health Centre, Vancouver, BC, Canada; 4 Department of Pathology and Laboratory Medicine, Faculty of Medicine, University of British Columbia, Vancouver, BC, Canada; 5 Department of Paediatrics, Faculty of Medicine, University of British Columbia, Vancouver, BC, Canada; University of Kwazulu-Natal, SOUTH AFRICA

## Abstract

**Introduction:**

Combination antiretroviral therapy (cART) can effectively prevent vertical transmission of HIV but there is potential risk of adverse maternal, foetal or infant effects. Specifically, the effect of cART use during pregnancy on mitochondrial DNA (mtDNA) content in HIV-positive (HIV+) women is unclear. We sought to characterize subclinical alterations in peripheral blood mtDNA levels in cART-treated HIV+ women during pregnancy and the postpartum period.

**Methods:**

This prospective longitudinal observational cohort study enrolled both HIV+ and HIV-negative (HIV-) pregnant women. Clinical data and blood samples were collected at three time points in pregnancy (13-<23 weeks, 23-<30 weeks, 30–40 weeks), and at delivery and six weeks post-partum in HIV+ women. Peripheral blood mtDNA to nuclear DNA (nDNA) ratio was measured by qPCR.

**Results:**

Over a four year period, 63 HIV+ and 42 HIV- women were enrolled. HIV+ women showed significantly lower mtDNA/nDNA ratios compared to HIV- women during pregnancy (p = 0.003), after controlling for platelet count and repeated measurements using a multivariable mixed-effects model. Ethnicity, gestational age (GA) and substance use were also significantly associated with mtDNA/nDNA ratio (p≤0.02). Among HIV+ women, higher CD4 nadir was associated with higher mtDNA/nDNA ratios (p<0.0001), and these ratio were significantly lower during pregnancy compared to the postpartum period (p<0.0001).

**Conclusions:**

In the context of this study, it was not possible to distinguish between mtDNA effects related to HIV infection versus cART therapy. Nevertheless, while mtDNA levels were relatively stable over time in both groups during pregnancy, they were significantly lower in HIV+ women compared to HIV- women. Although no immediate clinical impact was observed on maternal or infant health, lower maternal mtDNA levels may exert long-term effects on women and children and remain a concern. Improved knowledge of such subclinical alterations is another step toward optimizing the safety and efficacy of cART regimens during pregnancy.

## Introduction

An estimated 17 million women are infected with HIV (HIV+) worldwide [1]. Without intervention, vertical transmission rates during and after pregnancy range from 14% to 48% [2]. Since 1994, the use of antiretroviral therapy (ART) has contributed substantially to the prevention of vertical transmission [3] and with progression to the use of combination ART (cART), in conjunction with comprehensive obstetrical care, dramatically decreased neonatal infection rates (~0.4%) have been widely reported [4–10]. However, there is limited knowledge or data available on the long term safety of cART exposure in human foetuses during pregnancy [11–16], and how *in utero* cART exposure may affect HIV exposed uninfected (HEU) infants later in life.

cART continues to be the standard of care for pregnant women living with HIV, but as an increasing number of drugs are introduced and used in HIV treatment regimens, there are numerous examples of clinical cases where, for various reasons, regimens not well studied in pregnancy must be used. Although most individuals taking cART have tolerable and relatively mild side effects, it has also been associated with moderate or severe complications [17], particularly in pregnancy [18]. In addition to other suggested mechanisms [19–21], off-target effects of nucleoside reverse transcriptase inhibitors (NRTI) on the mitochondria and its polymerase-gamma [5, 22–25] are likely playing a role in many cART-induced toxicities. These can result in mtDNA depletion, increased mtDNA mutations, decreased mitochondrial gene expression, increased mitochondrial oxidative stress, and ultimately, mitochondrial dysfunction [19, 20, 26–31].

HIV+ ART-exposed women have higher rates of adverse perinatal outcomes compared to HIV- ART-unexposed women. Specifically, the incidences of preterm birth and being small for gestational age (GA) are significantly increased among infants born to HIV+ women taking ART during pregnancy compared to infants born to HIV- women [13, 16, 32–43], although the contribution of HIV, other cofactors and/or antiviral therapy remains unclear. Signs of mitochondrial dysfunction in infants, such as transient hyperlactatemia, are common [11]. Furthermore, altered blood mtDNA levels [11, 13–15], as well as increased blood mtDNA somatic mutations [29] have been reported in ART-exposed HEU infants. Somatic mtDNA mutations have been associated with aging and age-related diseases [44–46], including mitochondrial aging in the context of HIV and NRTI exposure [30]. Clinical symptoms suggestive of mitochondrial dysfunction in HEU infants born to HIV+ women treated with cART in pregnancy are either not [11,13,14,43], or rarely [47,48] observed. However, subclinical mitochondrial alterations in HIV+ mothers or their HEU infants could impact health later in life.

There have been conflicting reports regarding the effect of HIV and ART use during pregnancy on mtDNA levels. Observational studies comparing mtDNA levels in tissues from HIV+ ART-exposed pregnant women and their infants to HIV- women and their infants have reported higher, lower, or no significant change in levels of mtDNA [11, 13–15, 49–53]. The disparity between these findings may be related to differences in cART regimen type, cART duration between studies and/or the fact that multiple mechanisms may be at play. For example, exposure to some NRTI may induce mtDNA depletion [25] or increase mtDNA mutations, possibly through replication errors by polymerase gamma or oxidative damage [54] which may in turn induce mitochondrial biogenesis as an adaptive mechanism, favouring clonal expansion of mtDNA mutations [55–56]. Concurrently, other ART agents belonging to other drug class are reported to modulate autophagy [57–58], and can thereby affect mtDNA levels. There clearly is a need for further research using larger samples and rigorous study designs. Indeed, most studies conducted to date are cross-sectional, of small to moderate sample size, with often heterogeneous cART regimens. No previous study has assessed longitudinal mtDNA/nDNA ratio during pregnancy in HIV- or HIV+ cART treated women. Ultimately, understanding the pharmacopathology of cART is important because many ART drugs can cross the placenta, and thus may impact foetal development or long term health.

We aimed to establish whether subclinical mitochondrial alterations occur during pregnancy in HIV+ cART treated women by investigating peripheral blood mtDNA levels during and after pregnancy in HIV+ cART-exposed women, as well as in HIV- control women. This information is vitally important for ongoing global efforts to determine the safest and most effective application of cART in pregnancy.

## Materials and Methods

### Design

This single-site, prospective longitudinal observational study consisted of two cohorts: (i) HIV+ women (N = 65) using cART during pregnancy (cART started pre-conception or during pregnancy); (ii) HIV- women (N = 45). The Clinical Research Ethics Review Board at the University of British Columbia, Canada approved this study (H04-70540).

### Recruitment

All women were approached to participate from December 2004 to October 2008. The cohort of HIV+ women (N = 65) was recruited from the Oak Tree Clinic, a provincial referral centre in Vancouver, British Columbia (BC), Canada, which coordinates HIV care for all known HIV+ pregnant women in BC. The cohort of HIV- women (N = 45) were recruited from the same city.

During recruitment of controls, a deliberate effort was made to approach potential participants with similar characteristics to the HIV+ group. While this approach worked well toward reducing confounding factors (i.e. maternal age, smoking, alcohol consumption, and illicit drug use during pregnancy) between the groups, we did not achieve similar ethnicity among participants in the two groups due to cultural and ethical barriers encountered when dealing with small communities in which participation in an HIV study is negatively perceived. However, the ethnic distribution in our control sample was more closely comparable to the HIV+ group than the background Canadian population.

### Data Collection

At enrolment, demographic, behavioural and clinical parameters were collected. All participants provided written informed consent. Peripheral blood was collected from HIV+ participants at three time points in pregnancy (13-<23 weeks, 23-<30 weeks, 30–40 weeks), at delivery, and six weeks postpartum. Blood samples for mtDNA assays were collected in conjunction with clinically indicated blood tests for prenatal care, evaluation of HIV status and toxicity of cART. Clinical data pertaining to the infants born to HIV+ mothers was collected during the first week of life and at six weeks of age. HIV- pregnant women were asked to contribute peripheral blood samples during the same three time points during pregnancy to provide a reflection of physiological mtDNA dynamics in pregnancy. Peripheral blood was not collected from the control group at delivery or six weeks postpartum due to impracticalities in obtaining specimens.

### Mitochondrial DNA Assay

Peripheral blood was collected in ACD solution A tubes, transferred to a cryotube, and stored frozen at -80° C until used. Total DNA was extracted from 0.1 ml of whole blood using the QIAGEN DNA isolation kit, according to the manufacturer’s protocol and as previously described [25]. Mitochondrial gene cytochrome c oxidase subunit I and the nuclear gene for the accessory subunit of the polymerase gamma were quantified in duplicate by monochrome real-time quantitative PCR with fluorescent probes, using a Roche LightCycler 480. A standard curve was prepared with serially diluted plasmid DNA containing the two genes of interest. Both genes were quantified separately in duplicate on the same 96 well plate along with two internal controls, for a total of 20 samples per run. Results are expressed as the relative ratio between mtDNA and nDNA, according to the standard curve. Samples were assayed in a blinded and randomized fashion, but all extracts from a given participant were always assayed within a given run to minimize intra-subject variability. The intra- and inter-run coefficients of variation were5% and 10%, respectively.

### Data Analysis

Descriptive statistics report one standard deviation (SD) or standard error on the mean (SEM). To explore the effect of cART and HIV infection on the mtDNA ratios in pregnant women, a multivariate linear nested mixed-effects model was applied. In addition to the effect of HIV infection (HIV- *vs*. HIV+), the effect of the following was analysed: (a) maternal age at birth, (b) ethnicity (c) GA at time of sampling, (d) smoking, (e) alcohol consumption, and (f) use of illicit drugs (e.g. cocaine, heroin, see Table 1 footnote) throughout pregnancy (e.g. women reported active substance use at three or more time points in pregnancy). As platelets contain a small amount of mtDNA, platelet count was included in all models as a covariate. Women with missing data were casewise removed resulting in 265 mtDNA measures in pregnancy from 105 women.

**Table 1:** Demographic, clinical and laboratory characteristics of study participants (n = 105).

Characteristic	Mean ± SD or n (%)		
	HIV+	HIV-	p-value^b^
	(*n* = 63)	(*n* = 42)	
**Maternal Age (years)**	30 ± 6	31 ± 5	0.23
**Maternal Ethnicity**			0.0004
Aboriginal	18 (29)	5 (12)	
Caucasian	23 (37)	27 (64)	
Black	12 (19)	0 (0)	
Asian/Other	10 (16)	10 (24)	
**HCV Antibody+**			<0.0001
Positive	17 (27)	2 (5)	
Negative	40 (63)	16 (37)	
Unknown	6 (10)	24 (57)	
**HCV PCR+**			
Positive	8 (13)	1 (2)	0.0004
Negative	41 (65)	18 (43)	
Cleared	3 (5)	1 (2)	
Unknown	11 (17)	22 (52)	
**Substance use throughout pregnancy** ^**a**^			
Smoking	29 (46)	17 (40)	0.72
Alcohol	2 (3)	3 (7)	0.39
Illicit Drugs	6 (10)	7 (17)	0.43
**Platelets (10** ^**9**^ **/L)**			
13-<23 weeks	242 ± 65	253 ± 83	0.48
23-<30 weeks	257 ± 79	241 ± 72	0.27
30–40 weeks	257 ± 76	225 ± 62	0.03
Delivery	245 ± 75	-	-
Postpartum	276 ± 108	-	-

^a^ Substance use is defined as self-reported use of substance at ≥3 study visits. Illicit Drugs = heroin, cocaine, opioids, amphetamines, benzodiazepenes and/or MDMA (ecstasy).

^b^ P-values are from Fisher’s exact tests for categorical data, and t-tests for continuous data (Platelets

Stepwise model selection was used to remove any non-significant variables from the model, resulting in an optimal model containing only variables significantly associated with mtDNA/nDNA ratio. Significance of the variables was assessed using Chi-square tests comparing the log-likelihoods of a model containing the variable versus one with it removed. A significant test (p < 0.05) means that inclusion of the variable significantly increased the fit of the model. Where applicable, post-hoc comparisons between groups were conducted using Tukey tests modified for mixed-effects models as implemented in the multcomp package in R [54]. Adjusted p-values are reported.

To explore differences between HIV+ women, modelling procedures used were the same as those described above, with the addition of three HIV specific variables: (a) lifetime ART exposure, (b) duration of cART in pregnancy, and (c) CD4 nadir, selected as a proxy to indicate severity of HIV disease. After removal of missing data, there were 253 mtDNA measures distributed amongst 63 women. To further investigate the effects of cART exposure on mtDNA ratio, the percent change in mtDNA/nDNA ratios from delivery to postpartum were compared between women who remained on cART and those who stopped using cART after delivery, using ANOVA.

Comparisons of demographic, clinical and laboratory characteristics between HIV+ and HIV- women were made. Continuous variables were tested for differences between the groups using t-tests; Apgar scores were compared using a Wilcoxon rank-sum test; categorical variables were compared using Chi Squared tests or Fisher exact tests where appropriate.

## Results

### Participant Characteristics

In total, 105 pregnant women were enrolled in this study: 63 HIV+ pregnant women and 42 HIV- control pregnant women. Of the 115 women who were approached to participate, ten declined, corresponding to an enrolment rate of 91%. Demographic characteristics for both groups are summarized in Table 1, and HIV-specific characteristics of the HIV+ group are summarized in Table 2. Distribution of ethnicities was significantly different between the two groups (p = 0.0004) with more Aboriginal and Black/African women in the HIV+ group and more Caucasian and Asian women in the HIV- control group. The proportion of women reporting substance use (i.e., smoking, alcohol, illicit drugs) during pregnancy did not differ significantly between the two groups.

**Table 2:** HIV-specific clinical characteristics of HIV+ cohort (n = 63).

Characteristic	Median (range) or n (%)
**Time since HIV diagnosis (yrs)**	4 (0.2–17)
HIV diagnosis during index pregnancy	15 (21)
**CD4 Nadir (cells/μL)**	250 (20–910)
**Lifetime ART exposure (weeks)**	36 (2–800)
**cART exposure in pregnancy (weeks)**	20 (2–42)
**Timing of cART Initiation**	
Pre conception	17 (27)
2–23 weeks gestation	30 (48)
23-<30 weeks gestation	12 (19)
30–37 weeks gestation	4 (6)
**Immunovirological parameters at first visit (13–22 weeks)**	
CD4 (cells/μL)	445 (90–1200)
HIV viral load (log_10_copies/mL)	1.7 (0–3.8)
**Immunovirological parameters at delivery (32–40 weeks)**	
CD4 (cells/μL)	450 (100–1300)
HIV viral load (log_10_copies/mL)	0 (0–3.9)
**Vertical transmission of HIV**	0 (0)

### cART Regimens

HIV+ women treated during pregnancy were on a variety of regimens based on their individual circumstances (Please see Table 3 for detailed information). These included 56 women receiving a regimen containing ZDV and lamivudine (3TC); 59 women receiving a one or more protease inhibitors (PI) [n = 36 on nelfinavir (NFV), n = 28 on ritonavir-boosted lopinavir (LPV/r), n = 4 on ritonavir-boosted atazanavir (ATV)]; and 8 women receiving a non-NRTI (NNRT1)-containing regimen [n = 6 on nevirapine (NVP), n = 2 on efavirenz (EFV)].

**Table 3:** Combination antiretroviral therapy regimens taken during pregnancy by HIV positive study cohort (n = 63).

NRTI	N	NNRTI	N	PI	N
**ZDV/3TC**	49	—-	42	NFV	24
				LPV/r	14
				NFV/LPV/r	4
		NVP	5	—-	2
				NFV	2
				LPV/r	1
		EFV	2	—-	1
				LPV/r	1
**ZDV/3TC/TDF**	3	—-	3	NFV	1
				LPV/r	1
				NFV/LPV/r	1
**ZDV/3TC/TDF/ABC**	1	—-	1	ATV/LPV/r	1
**ZDV/3TC/TDF/DDI**	1	—-	1	ATV/LPV/r	1
**ZDV/3TC/ABC**	2	—-	2	—-	1
				NFV/LPV/r	1
**ZDV/DDI**	1	—-	1	NFV	1
**3TC/TDF**	1	—-	1	ATV/r	1
**d4T/3TC**	3	—-	3	NFV	1
				LPV/r	2
**FTC/TDF**	2	—-	1	ATV/r	1
		NVP	1	LPV/r	1
**Total N**	**63**		**8**		**59**

NRTI, nucleoside reverse transcriptase inhibitor; NNRTI, non-nucleoside reverse transcriptase inhibitor; PI, protease inhibitor; ZDV, zidovudine; 3TC, lamivudine; TDF, tenofovir; ABC, abacavir; ddI, didanosine; d4T, stavudine; FTC, emtricitabine; NVP, nevirapine; EFV, efavirenz; NFV, nelfinavir; LPV, lopinavir; NFV, nelfinavir; ATV, atazanavir; r, ritonavir boosted

### Infant Outcomes

Infants born to HIV- and HIV+ women were not significantly different in terms of GA at birth, sex, birth weight, birth length, Apgar score at five minutes, and frequency of congenital anomalies and neonatal morbidities (Table 4). The proportion of preterm births observed in this population (18% for HIV+ women, 17% for HIV- women; Table 4) was higher than the provincial rate for preterm births (~5.3) [59] but not different between the groups. None of the 63 infants born to HIV+ women in this study were infected with HIV.

**Table 4:** Perinatal and neonatal outcomes for HIV+ (n = 63) and HIV- (n = 42) maternal infant pairs.

Characteristic	Mean ± SD (range) or n (%)		
	HIV+	HIV-	p-value
	(*n* = 63)	(*n* = 42)	
**Live birth frequency** ^**a**^	63 (100)	41 (98)	0.41
Proportion singleton births	63 (100)	42 (100)	-
Proportion male (%)	32 (51)	22 (52)	1
**GA at birth (weeks)**	39 ± 2 (32–42)	39 ± 2 (34–42)	0.49
Preterm birth (<37 weeks)	11 (18)	7 (17)	1
**Birth weight (g)**	3078 ± 484 (1800–4075)	3095 ± 557 (1925–4535)	0.87
**Birth length (cm)**	50 ± 4 (35–57)	50 ± 4 (38–57)	0.53
**Median Apgar score at 5 min (range)**	9 (7–10)	9 (8–10)	0.31
**Congenital abnormality** ^**c**^	4 (3)	1 (3)	0.65
**Neonatal complications** ^**d**^	6 (9)	3 (8)	0.24
**Mild/Moderate neonatal withdrawal symptoms**	13 (19)	7 (18)	0.8
**Neonatal ZDV exposure** ^**b**^	62 (100)	2 (5)	-
**Neonatal NVP exposure** ^**b**^	9 (14)	2 (5)	-

^a^ One infant born to an HIV- mother was stillborn.

^b^ Two HIV- control women were considered at high risk of contracting HIV due to drug use and unprotected intercourse with partners with unknown HIV status; therefore, infants were treated with zidovudine (ZDV) and nevirapine (NVP) as per standard of care.

^c^ Maternal HIV+ group: pulmonary artery stenosis (n = 2), pyloric stenosis (n = 1), hydronephrosis (n = 1); Maternal HIV- group: hydronephrosis (n = 1).

^d^ Maternal HIV+ group: mild respiratory distress (n = 1), respiratory syncytial virus (RSV) infection (n = 1), seizures (n = 1), sepsis (n = 1), apnoea of prematurity (n = 1), pneumonia (n = 1); Maternal HIV- group: mild respiratory distress (n = 1), neonatal intensive care unit for >24 hours (n = 1), hyperbilirubinaemia (n = 1).

Continuous variables were tested for differences between the groups using t-tests; Apgar scores were compared using a Wilcoxon rank-sum test; categorical variables were compared using Chi Squared tests or Fisher exact tests where appropriate.

SD, standard deviation

### mtDNA/nDNA ratios in HIV+ compared to HIV-

**Fig 1:**
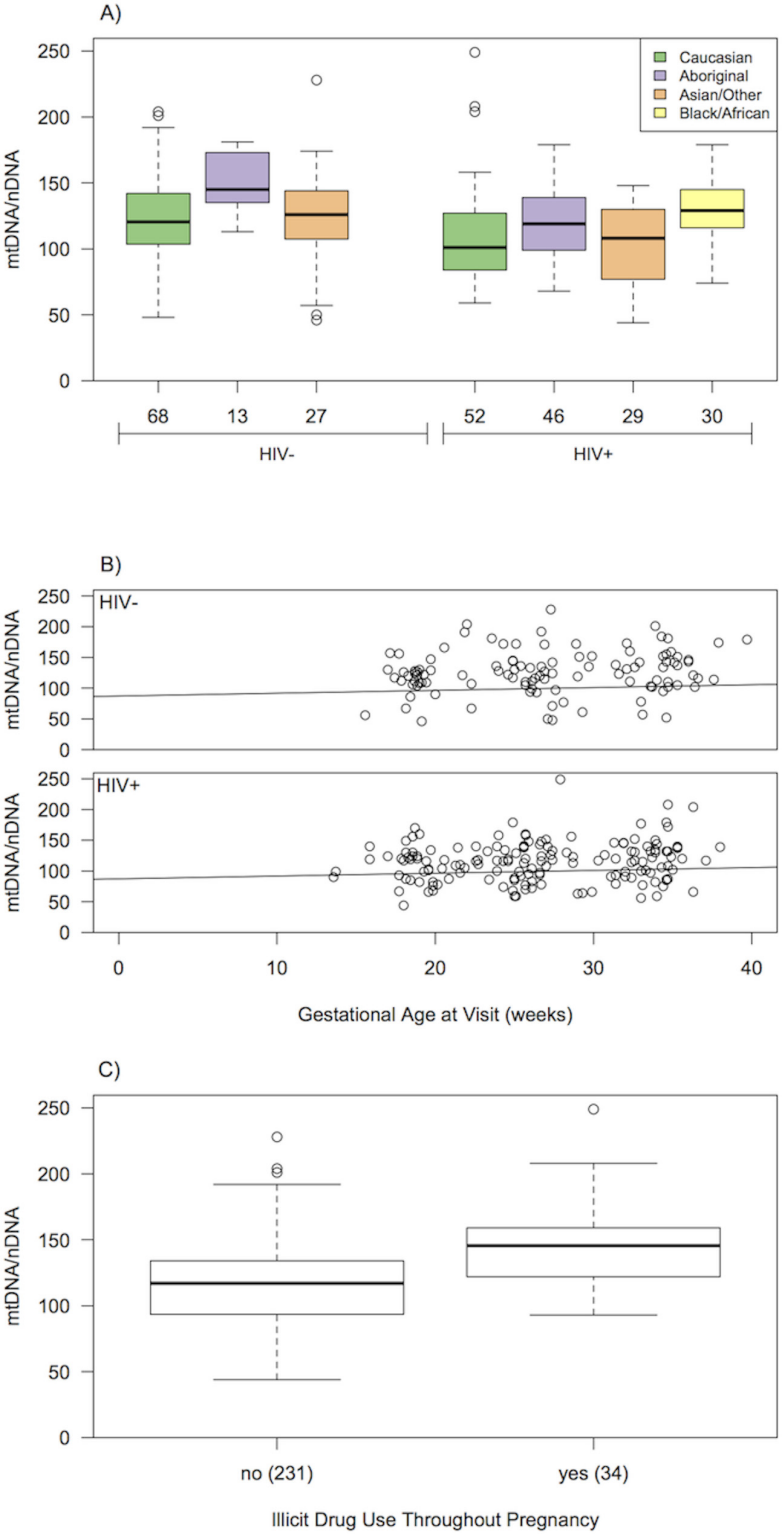
Significant associations between mtDNA/nDNA ratio and variables of interest for both HIV- and HIV+ cohorts (*N* = 105). (A) Ethnicity, (B) GA at visit (weeks) and (C) Illicit drug use. In A and C, the horizontal line in the boxplots indicates the median value, boxes represent the interquartile range, whiskers indicate 1.5 times the interquartile range, while points indicate outliers. In B, the best-fit line from the mixed-effects model controlling for platelets and other significant variables is shown. All samples were collected during pregnancy.

The within woman intra-class correlation from the mixed-effects model was estimated at 0.73, thus measurements of mtDNA/nDNA ratio in the same woman at different times were highly correlated. During pregnancy, the mtDNA/nDNA ratios within each woman were relatively stable over time with a slight upward trend. Four variables were significantly associated with mtDNA/nDNA ratio after controlling for repeated measurements on women and platelet count in the multivariable mixed-effects model: HIV infection (p = 0.003), ethnicity (p = 0.024), GA (p = 0.02), and illicit drug use (p = 0.004; Table 5, Fig 1A–1C).

**Table 5:** Means, model estimates, and log-likelihood ratio test Chi-squared results for the mixed-effects modelling of mtDNA levels in all HIV+ and HIV- pregnant women (n = 105).

	Mean (SEM)^a^	Estimated Model coefficient (±SEM)^b^	LRT^d^ (df)	p-value
**Intercept**		87.2		
**Ethnicity**			9.5 (3)	0.02
Caucasian	117 (7)	reference		
Asian/Other	112 (10)	-3.0 (7.2)		
Black/African	129 (10)	26.8 (9.5)		
Aboriginal	126 (4)	1.2 (7.7)		
**HIV status**			9.1 (1)	0.003
HIV-	126 (3)	reference		
HIV+	115 (3)	-18.0 (6.1)		
**GA at visit (weeks)**		0.5 (0.2)	5.4 (1)	0.02
**Illicit drug use** ^c^			8.1 (1)	0.004
No	115 (2)	reference		
Yes	147 (6)	24.5 (8.7)		

^a^ Means (± SEM) are reported for the raw data without correction for covariates.

^b^ Estimated effects after taking covariates into account.

^c^ The modelling did not differentiate between women using 2 or more illicit drugs during pregnancy and women only taking one illicit drug.

^d^ LRT = likelihood-ratio test statistic

SEM, Standard error on the mean

df, degrees of freedom

GA, gestational age

The HIV+ group had significantly lower mtDNA/nDNA ratios than the HIV- control group during pregnancy (estimated model coefficients, estimated effect = -18.0 ± 6.1 SE, p = 0.003; Table 5). Black/African women had significantly higher ratios compared to Asian/other (p = 0.02) and Caucasian women (p = 0.02), as shown by post-hoc Tukey-tests. Increasing GA (slope = 0.46 ± 0.20 SE, p = 0.02) and reported illicit drug use (estimated effect = 24.5 ± 8.7 SE, p = 0.004) were associated with an increase in mtDNA/nDNA ratio (Table 5). The distribution of illicit drug users was uneven in respect to ethnicity and HIV status, as women of Black/African ethnicity had no self-reported incidences of illicit drug use during pregnancy, but were more likely to be HIV+. Although Black/African women had higher mtDNA/nDNA ratios on average, and were over-represented in the HIV+ group compared to the other ethnicities, there was still a strong negative effect of HIV status on mtDNA/nDNA ratio after ethnicity and drug use were taken into account. These results may thus be conservative in estimating the difference in mtDNA/nDNA ratio between HIV+ and HIV- pregnant women.

### mtDNA/nDNA ratios among HIV+ women

**Fig 2:**
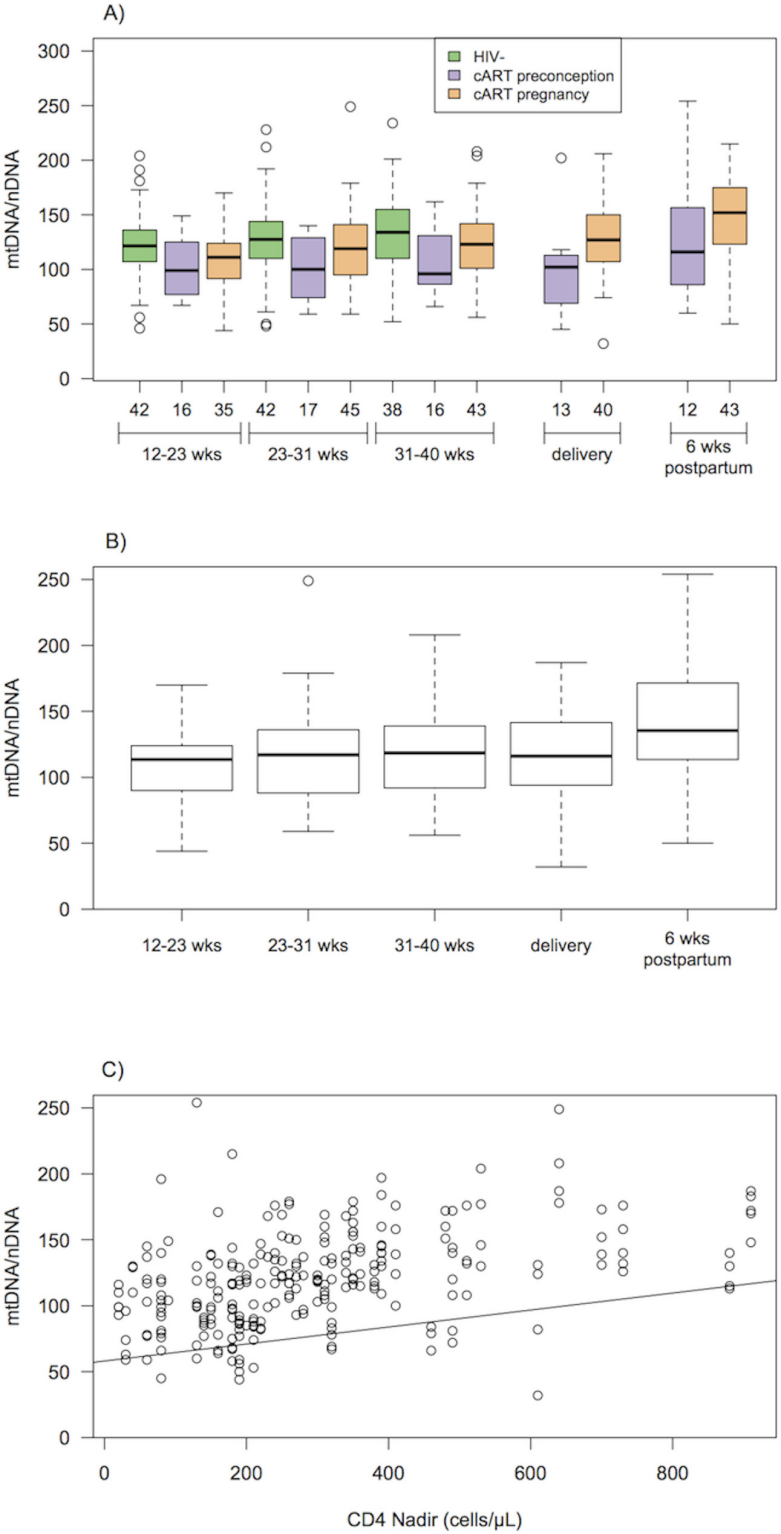
Temporal variation in mtDNA/nDNA ratio during pregnancy, at delivery and postpartum for the HIV+ cohort. A) mtDNA/nDNA ratio in HIV- women (n = 42), HIV+ women who initiated cART prior to conception (n = 17) and continued throughout pregnancy, and HIV+ women who initiated cART during pregnancy (n = 46) at each sample time point (13–22 weeks, 23–30 weeks, 31–40 weeks, delivery, 6 weeks postpartum); mtDNA/ nDNA ratio among HIV+ women (n = 63) for the significant variables of (B) time of visit (13–22 weeks, 23–30 weeks, 31–40 weeks, delivery, 6 weeks postpartum), where the horizontal line in the boxplots indicates the median value, boxes represent the interquartile range, whiskers indicate 1.5 times the interquartile range, while points indicate outliers; (C) CD4 nadir, where the best-fit line from the mixed-effects model is shown.

Among the HIV+ women, the within woman intra-class correlation from the mixed-effects model was 0.44, indicating that measurements in the same woman were somewhat correlated. While there appeared to be a trend toward lower mtDNA/nDNA ratios among women who started cART therapy pre-conception (n = 17) versus those who started cART during pregnancy (n = 46), after other significant variables were taken into account, there were no significant differences between these two groups in terms of mean mtDNA/nDNA ratios over time (p = 0.67; Fig 2A). Three variables were significantly associated with mtDNA/nDNA ratio after controlling for repeated measurements and platelet count in the multivariable mixed-effects model: timing of sample (pregnancy, delivery and postpartum; p<0.0001), ethnicity (p = 0.03), and CD4 nadir (p<0.0001; Fig 2B–2D).

When post-hoc comparisons were conducted, only comparisons between samples taken during pregnancy versus postpartum showed significant differences in mean mtDNA/nDNA ratio. Samples taken at 13-<23 weeks, 23-<30 weeks, 30–40 weeks and delivery (p-values = <0.001, <0.001, 0.002, and <0.001 respectively, as shown by post-hoc Tukey tests) had lower mtDNA ratios compared to those taken postpartum. Comparisons among samples collected during pregnancy were not significantly different. HIV+ Black/African women had significantly higher mtDNA ratios than the Asian/other HIV+ women (p = 0.02) as shown by post-hoc Tukey tests. Higher CD4 nadir was positively associated with higher mtDNA/nDNA ratios (model coefficients, slope = 0.06 ± 0.02 SE; Table 6). The estimates and standard errors of the model coefficients, and the Chi-square and p-value for the tests of significance are summarized in Table 6.

**Table 6:** Means, model estimates, and Chi-squared results for the mixed-effects modelling of mtDNA levels within the 63 HIV+ pregnant women.

	Mean (±SEM)^a^	Estimated Model coefficient (±SEM)^b^	LRT^d^ (df)	p-value
**Intercept**		58.1		
**Ethnicity**			9.3 (3)	0.03
Caucasian	116 (4)	reference		
Asian/Other	106 (6)	-8.5 (8.8)		
Black/African	131 (4)	19.9 (8.4)		
Aboriginal	124 (4)	2.0 (7.5)		
**Timing of visit**			29.3 (4)	< 0.0001
13–22 weeks	111 (4)	reference		
23–30 weeks	114 (5)	-0.8 (4.5)		
31–40 weeks	119 (5)	3.9 (4.6)		
Delivery	117 (6)	-0.03 (4.7)		
6 weeks postpartum	139 (6)	20.5 (4.8)		
**CD4 Nadir**		0.06 (0.02)	17.1 (1)	< 0.0001

^a^ Means (± SEM) are reported for the raw data without correction for covariates.

^b^ Estimated effects after taking covariates into account.

^d^ LRT = likelihood-ratio test statistic

SEM, Standard error on the mean

df, degrees of freedom

### mtDNA/nDNA ratios among HIV+ women postpartum samples

Of the 63 HIV+ women, 28 remained on cART postpartum, 28 stopped cART postpartum, and postpartum cART information was not available for 7 women. Of these, 44 had data for mtDNA/nDNA ratios at both delivery and postpartum (20 who remained on cART, and 24 who discontinued). There was no significant difference in the percent change in mtDNA/nDNA ratio from delivery to postpartum between HIV+ women who remained on cART postpartum (mean percent change in mtDNA/nDNA ± SD ratio 13 ± 16%) compared to those women who did not (7 ± 18%; ANOVA, F = 0.99, p = 0.33).

## Discussion

Mitochondrial DNA levels through pregnancy have not been previously studied in a longitudinal design and the HIV- control group illustrates stability of levels within women through pregnancy with modest increase prior to delivery. Our study demonstrates the novel finding that compared to HIV- controls, HIV+ cART-exposed women had significantly lower blood mtDNA levels during pregnancy. This finding was remarkable in that the HIV+ group consisted of participants of ethnicities that, on average, have higher mtDNA ratios. This finding is consistent with other studies that have found significantly decreased mtDNA levels in other tissues such as umbilical cord blood [49,50] and placental tissue [50, 51] of HIV+ women compared to HIV- women. Of note, this is also consistent with another study [43], which reported non-significantly lower mtDNA levels shortly after delivery in HIV+ women treated with ART therapy compared to HIV- women. Higher mtDNA levels in infants born to HIV+ cART-treated mothers compared to HIV- women were previously reported by our group [11] and others [17,22], and may be a compensatory mechanism to overcome HIV/ART associated mitochondrial toxicity *in utero* [11,14].

It is critical to be aware of lower mtDNA levels during pregnancy because altered mtDNA may impact metabolism and energy production within the placenta, affecting foetal growth and development. Furthermore, mtDNA depletion during pregnancy may contribute to the neurological symptoms suggestive of mitochondrial dysfunction observed in some children born to HIV+ ART-exposed women [47, 48]. In this study, no infants born to HIV+ mothers showed clinically recognised neurological symptoms suggestive of mitochondrial dysfunction within the study period. There were instances of transient mild to moderate hyperlactatemia but no lactic acidosis. This is consistent with previous studies among similarly exposed infants. Nevertheless, lower mtDNA levels in HIV+ mothers may contribute to their children’s future development, growth and health. Further, there were no significant differences in obstetrical and neonatal outcomes of HIV+ and HIV- pregnancies in this study, which was surprising considering that many studies have shown a significantly increased incidence of preterm birth and low birth weight among infants born to HIV+ ART-exposed women compared to infants born to HIV- women [13,16,32–42]. However, HIV- women in this study were unusually well matched with respect to other risk factors known to be associated with prematurity/low birth weight, including smoking and illicit drug use illustrated by the high rates of preterm birth in both our study and control arms. This matching may also have exerted a negative effect on the HIV- women’s mtDNA/nDNA ratios compared to low risk HIV- women, minimizing any difference between their results and that of our HIV+ cohort.

In spite of a trend for women who initiated cART during pregnancy to have increased mtDNA levels compared to women who initiated therapy prior to conception, this effect was not statistically significant. While it is possible that timing of initiation and duration of cART could impact mtDNA levels, our study lacked the statistical power for such a sub-analysis and therefore could not adequately ascertain the effect of timing of cART initiation on mtDNA. Furthermore, women who were taking cART prior to conception likely had greater HIV disease duration and severity, as indicated by a lower CD4 nadir, something that could impact mitochondrial function in addition to exposure to cART. Indeed, the observation that lower CD4 nadir was positively associated with lower mtDNA/nDNA ratio among HIV+ women would be consistent with several studies that have found significant mtDNA depletion in the peripheral blood of untreated HIV+ adults compared to uninfected controls [21,60–62]. Whether cART initiation can fully reverse this effect is unknown.

Among HIV+ women, mtDNA levels were significantly higher postpartum than during pregnancy, both in HIV+ women who continued and those who discontinued cART. This result indicates that pregnancy/delivery itself is associated with a change in blood mtDNA levels. Postpartum peripheral blood samples in HIV- women would be required to tease apart the effects of cART, HIV, and pregnancy itself on mtDNA levels, which were not available in this study, but warrants further investigation. Despite our inability in this study to compare postpartum HIV+ levels to HIV- levels, we hypothesize that the metabolic stress of pregnancy through the high growth and development of the placental/fetal unit likely suppresses mtDNA/nDNA levels which appear to rebound post-partum.

### Study Strengths and Limitations

The longitudinal design of this study allowed a more extensive documenting of blood mtDNA levels during pregnancy among HIV+ cART-exposed women relative to cross-sectional studies. Furthermore, the fact that the HIV- and HIV+ groups were well matched with respect to important factors such as substance use allowed consideration of these possible confounders. However, these data have several limitations. Firstly, our analyses included peripheral blood but other tissues may show different mtDNA levels, limiting the interpretation of our results in the context of other studies that utilized different tissue types. Secondly, we could not distinguish between the impacts of HIV infection itself and that of cART on mtDNA levels because not offering cART to HIV+ pregnant women would be unethical. Nevertheless, this adds an important confounding factor to our results as both cART and HIV could independently impact mtDNA. Thirdly, the lack of peripheral blood samples at delivery and six weeks postpartum from HIV- women makes it difficult to interpret the increased mtDNA levels seen postpartum in HIV+ women. Finally, the groups were not balanced with respect to the proportions of ethnicities.

## Conclusions

This is the first study to assess temporal trends of mtDNA/nDNA ratios during pregnancy. In both HIV+ and HIV- women, mtDNA/nDNA was observed to be relatively stable throughout pregnancy; however HIV+ women had lower mtDNA/nDNA ratios compared to HIV- women after controlling for ethnicity and illicit drug use. In HIV+ women, higher CD4 nadir was positively associated with higher mtDNA/nDNA ratio, although this association was not influenced by cART. Though some data exist on this subject, pregnant HIV+ women receiving cART are still understudied and there is much need for research in this population. While cART successfully reduces the vertical transmission rate of HIV in infected pregnant women, further research is critical to improve optimal selection of the safest regimens in pregnancy.
